# Current status and capabilities of cancer prevention and treatment in county-level hospitals in Fujian Province: a baseline survey under the hierarchical medical system

**DOI:** 10.3389/fpubh.2026.1955931

**Published:** 2026-10-14

**Authors:** Chen Guo, Qingmei Feng, Zuotian Yu, Haipeng Xu

**Affiliations:** Department of Medical Administration, Clinical Oncology School of Fujian Medical University, Fujian Cancer Hospital, Fuzhou, China

**Keywords:** cancer prevention and treatment, county-level hospital, hierarchical medical system, medical resources, survey

## Abstract

This paper presents an overall estimate on the current status and capabilities of cancer prevention and treatment at selected county-level hospitals in Fujian Province (southeast China) to obtain baseline data for further development, construction, and reformation of county-level cancer prevention and treatment centers under the hierarchical medical and healthcare system. A province-wide survey was conducted by Fujian Cancer Hospital from June to August 2025 using a custom-made ordinary questionnaire of 40 questions to investigate cancer-specific medical resources and the comprehensive service capabilities of 35 targeted county-level hospitals in 33 counties. Effective feedback was reported by 33 participating hospitals in 31 counties. The results are analyzed via self-programmed Python codes. The following results are obtained: (a) Twenty-five participating county-level hospitals have no independent cancer-specific wards; 29 participating county-level hospitals face a shortage of medical staff; and medical devices can meet the demand of primary diagnosis and treatment; (b) Insufficient prevention and screening is reported: 16, 9, and 12 county-level hospitals have never conducted cancer risk assessment, high-risk group management, and cancer screening, respectively; (c) Full coverage of cancer multidisciplinary teams (MDTs) has yet to be achieved, and 8 hospitals have no comprehensive treatment standardization, regulation, or implementation; (d) Bidirectional referral is still ineffective due to lack of referral regulation, and 16 participating county-level hospitals have conducted fewer than 10 cases in the past year; (e) Cancer information platforms and cancer-specific databases are still in the early stages and need substantial and sustained effort. Further development of county-level cancer prevention and treatment centers is necessary to increase medical resources for cancer prevention and treatment, enhance cancer prevention and treatment capabilities, and promote the implementation of hierarchical diagnosis and treatment in cancer-specific fields.

## 1 Introduction

According to GLOBOCAN 2022, cancer is a major public health threat in the 21st century and causes almost 20 million cases and close to 10 million deaths worldwide (1). In the past 30 years, cancer has become the second leading cause of death in China (2). In 2022 alone, China reported 482.47 × 10^4^ incidences and 257.42 × 10^4^ deaths caused by cancers (3), which substantially increased compared with the 406.40×10^4^ incidences and 241.35 × 10^4^ deaths reported in 2016 (4). As a southeastern province of China, Fujian Province also faces severe increases in cancer incidence and death.

In the last 10 years, cancer has surpassed cardiovascular and cerebrovascular diseases as the leading cause of death in Fujian Province. In 2023, cancer accounted for 24.52 and 27.91% of total deaths in urban and rural areas, respectively, in Fujian Province (5). From 2011 to 2021, the cancer survival rate in Fujian Province slowly increased from 29.1 to 39.1% at a low level (5), and the age-standardized 5-year relative survival rates in rural and urban areas were 37.1 and 41.7%, respectively, with a difference of approximately 4.6% (5). A continuous study revealed that the age-standardized 5-year relative survival rate from 2021 to 2023 in urban areas (49.3%) was much higher than that in rural areas (37.9%) (6). Another study from 2012--2014 suggested that patients in urban areas have greater relative survival rates than those in rural areas do (32.34 and 31.29%) (7). Liver cancer (8), breast cancer for women (9), and cervical cancer (10) also suggest similar cancer survival gaps between rural and urban areas. The above lower age-standardized 5-year relative survival rate data in rural areas from 2011--2023 indicate a significant gap in medical resources and technological levels between rural and urban areas in Fujian Province. A similar gap can also be observed in the whole nation (4). Lower cancer survival rates in rural areas are also reported abroad. Cancer mortality in rural areas of America is reported higher than that in metropolitan area (11, 12), and screening, diagnosis, treatment, and follow-up in rural area of America are lower than those in urban area (13). The American Cancer Society has continuously reported in 2023 and 2025 that cancer survival rates of non-metropolitan areas are lower than that of metropolitan areas (14, 15).

To balance this gap, China has conducted ongoing medical and health system reform to develop a hierarchical medical system consisting of tertiary hospitals, secondary hospitals, and primary-level hospitals (16), and certain progress has been achieved in Fujian Province (17, 18). Tertiary hospitals are generally located in provincial capitals and prefecture-level cities, and focus on diagnosis of complex and difficult cases, as well as cases referred from lower-level hospitals. Secondary hospitals are generally located in counties, and focus on clinical diagnosis and standardized treatment of common diseases, as well as conducting screening and bidirectional referral. Primary-level medical institutions are generally located at towns and villages, and focus on prevention and intervention on chronic diseases, as well as corresponding health record setting, follow-up, science popularization, and regular physical examination. One of the key aims of hierarchical medical systems is to enhance the ability of county-level hospitals to meet the demands of local cancer patients with different diagnoses and treatment needs by improving the bidirectional referral mechanism, medical service availability, and standardization level of diagnosis and treatment.

County-level hospitals, as the core of the rural medical and health service system, determine the capacity for cancer prevention and treatment in rural areas. However, county-level hospitals in Fujian Province generally face obvious inadequacies in terms of the use of cancer-specific devices and personnel, such as insufficient in-patient beds, doctors, and high-end medical devices, which would be illustrated in Section 3.1. These problems lead to low standardization and homogenization of cancer diagnosis and treatment for county-level hospitals and further decrease the cancer survival rate in rural areas of Fujian Province. Device and personnel shortages of county-level hospitals are also reported in other provinces in China (19), as well as in America (20), Korea (21), and Nigeria (22). Therefore, improving the ability of county-level hospitals for cancer screening, early diagnosis, standardized treatment, and regional collaborative management is crucial and urgent (23).

However, detailed information on such inadequacies is still insufficient in Fujian Province. To this end, this paper conducts a survey aimed at analyzing the current status of medical resources and the capability of cancer diagnosis and treatment in county-level hospitals. To conduct diagnosis and treatment of high-incidence cancers, such as thoracic tumors, gastrointestinal tumors, breast tumors, and so on, a county-level hospital should be equipped with corresponding medical resources, including medical personnel (such as doctors and nurses) and medical devices (such as X-ray, spiral CT, MRI, and so on). Moreover, diagnosis and treatment of high-incidence cancers would also be greatly affected by comprehensive service capabilities of a county-level hospital, such as prevention, nursing, collaboration, informatization, and so on. Therefore, we should investigate the current status of medical resources and comprehensive service capabilities of county-level hospitals in Fujian province to grasp a general landscape to summarize the weaknesses of current medical system and provide a reliable ground for further development, reformation, and decision-making of cancer prevention and treatment in county-level hospitals. With the survey data, a potential target for narrowing the urban–rural medical gap, enhancing county-level cancer prevention and control capabilities, and reducing the disease burden on cancer patients in rural areas might be foreseen.

## 2 Materials and methods

### 2.1 Survey purpose in county-level hospitals

From June to August in 2025, Fujian Cancer Hospital conducted a comprehensive survey to determine the current capabilities of cancer prevention and treatment at county-level public hospitals in Fujian Province, Southeast China. As discussed in the previous section, this survey investigates a general landscape of current status of medical resources and comprehensive service capabilities in county-level hospitals in Fujian province. The survey is province-wide by accessing 33 of all 53 counties in 8 of 9 prefecture-level cities in Fujian Province to reach 62.3% county coverage. The remaining prefecture-level city is a special economic zone, and has no county or county-level city under its jurisdiction, so that is not included in our survey. Among these 33 targeted counties, 34 county-level hospitals were selected from Fujian Province as part of the “Thousand County Project” in China (24, 25), indicating that at least one county-level hospital was selected from each targeted county. Moreover, two more hospital outside of the “Thousand County Project” list volunteered to participate in our survey. A total of 36 homemade ordinary questionnaires were issued, 33 of which were collected and found to be effective, with a 94.3% response rate to cover 31 of 33 targeted counties, including 10 tertiary hospitals and 23 secondary hospitals. Three hospitals did not provide feedback questionnaires. The distribution of participating hospitals is shown in Table 1, where the numbers within the parentheses denote the quantity of counties. Among the 33 county-level hospitals included in our survey, 31 were within the menu of the “Thousand County Project,” and the remaining two volunteered to participate.

**Table 1:** Distribution of 33 participating hospitals.

City	Issued questionnaires (targeted counties)	Collected questionnaires (participating counties)	Tertiary hospital	Secondary hospital
Fuzhou	5 (5)	5 (5)	1	4
Zhangzhou	4 (4)	4 (4)	1	3
Quanzhou	9 (7)	8 (6)	5	3
Sanming	6 (6)	6 (6)	0	6
Putian	1 (1)	1 (1)	1	0
Nanping	3 (3)	3 (3)	1	2
Longyan	4 (4)	3 (3)	0	3
Ningde	3 (3)	3 (3)	1	2
Total	35 (33)	33 (31)	10	23

### 2.2 Survey design and participants

A custom-made questionnaire was used to collect the survey data. The questionnaire was designed by the Expert Committee of the Cancer Prevention and Treatment Center of Fujian Province, which consists of senior doctors of high-incidence cancer categories, veteran medical technologists, and seasoned hospital administrators. During the early discussion and design of questionnaire, the expert committee found that county-level hospitals in Fujian province generally had a low level of informatization and were unable to provide accurate statistical data according to cancer types. Thus, for a tradeoff between data accuracy and statistical labor, the questionnaire consists of a total of 40 questions to sketch a landscape of two main aspects of county-level hospitals: (1) medical resource allocation and (2) cancer-related comprehensive service capabilities. Both aspects can be divided into more subcategories, as elaborated in the result section.

The questionnaire participants were the heads or oncology directors of the 35 selected county-level hospitals. All the participants were employed full-time at the hospital and were willing to participate and complete the survey. To guarantee precision, questionnaires were handed out one by one to the participants in each hospital. A detailed introduction to the questionnaire was given to the participants, and the necessary questionnaire training was provided. To ensure valid feedback, feasible and flexible communication channels supported by WeChat software, email, and cellphones were established between the participants and the Expert Committee for any questionnaire enquiry. The questionnaire had been assessed by 6 independent experts of cancer diagnosis and treatment via I-CVI (Item-level Content Validity Index). The results suggest all items of our questionnaire score over 0.78 and S-CVI/Ave (Scale-level Content Validity Index (average)) scores 0.917 to ensure data reliability.

### 2.3 Data analysis

Statistical analysis was performed via the programming code Python (packages Numpy and Matplotlib). Descriptive statistical methods are adopted to organize, summarize, and present the data to convey the central tendency, dispersion, and distribution.

## 3 Results

### 3.1 Medical resource allocation in participating county-level hospitals

#### 3.1.1 Availability of oncology departments and in-patient beds

The 33 effective questionnaires show that the diagnosis and treatment specialties of 27 participating county-level hospitals include the oncology discipline. Among the 27 participating county-level hospitals, 19 out of 27 have set up oncology wards with cancer-specific in-patient beds. Unlike higher-level hospitals, 11 out of these 19 county-level hospitals mix oncology wards with other medical departments, while the remaining 8 hospitals have better medical facilities and relatively more cancer patients to establish independent oncology wards. The availability of the oncology department and ward is shown in Figure 1.

**Figure 1:**
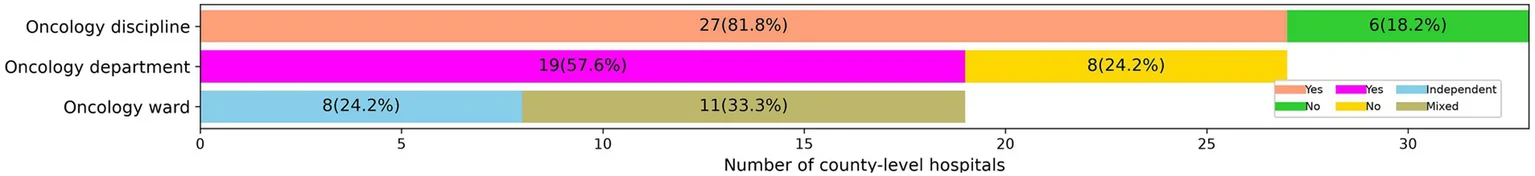
Availability of oncology discipline, department, and ward in 33 participating hospitals.

Figure 2 shows the number of cancer-specific in-patient beds of participating county-level hospitals from most to least common. The 19 (57.6%) participating county-level hospitals with independent oncology wards and mixed wards are distinguished by the same colors in Figure 1 into two groups. We can see that county-level hospitals with independent oncology wards generally have more in-patient beds than do those with mixed wards.

**Figure 2:**
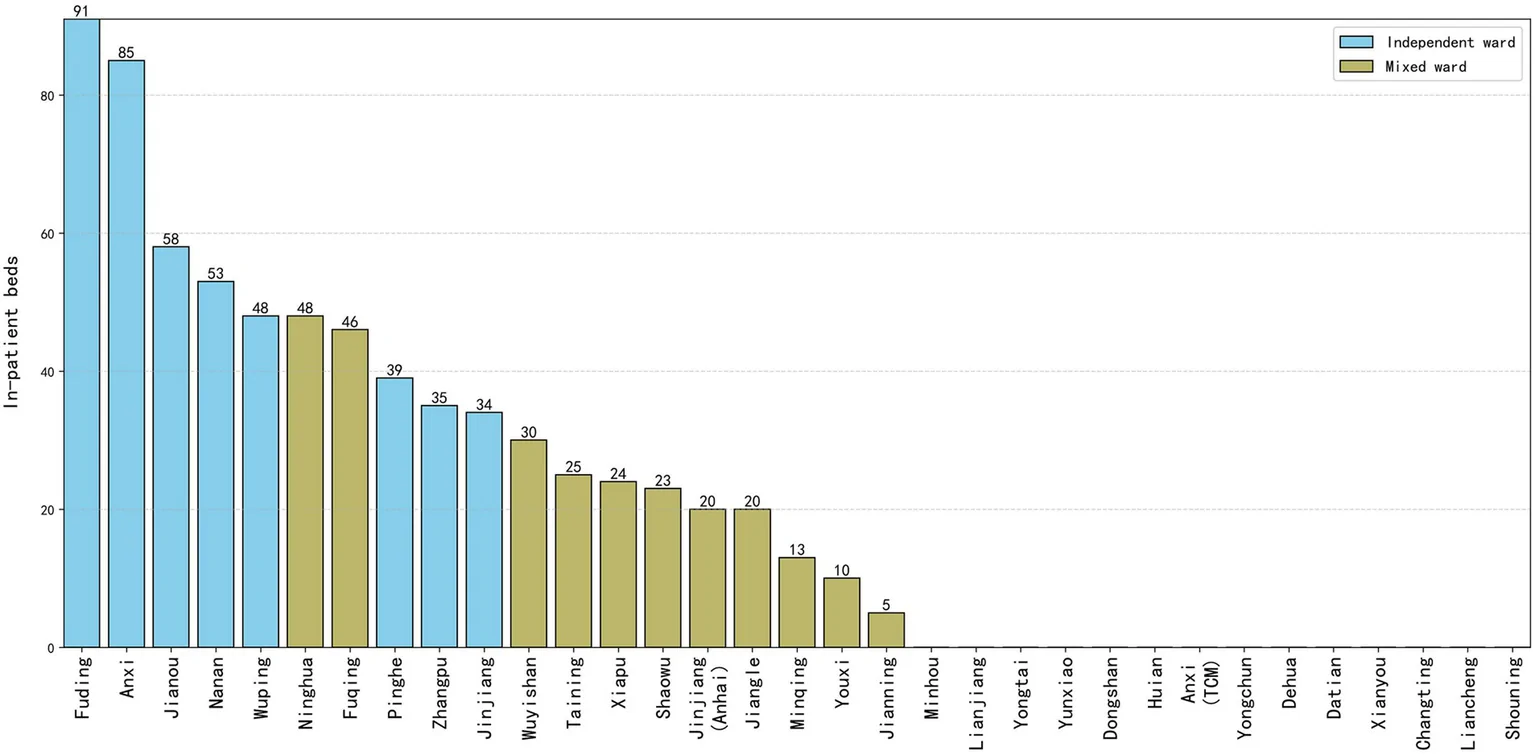
Cancer-specific beds in participating county-level hospitals.

#### 3.1.2 Cancer-specific medical personnel

We convert cancer-specific medical personnel in participating county-level hospitals by referring to the requirement of tertiary and secondary oncology hospitals in China that at least 0.4 nurses and a minimum doctor–nurse ratio of 1:1.6 should be staffed (26). However, our survey data suggest a significant insufficiency of oncologists and nurses in county-level hospitals in Fujian Province (see Figure 3). In Figure 3, the number on the horizontal axis denotes the quantity of cancer-specific beds. Figure 3 shows that only 4 (12.1%) participating county-level hospitals simultaneously had sufficient oncologists and nurses, whereas only 8 (24.2%) additional participating county-level hospitals had sufficient cancer-specific nurses.

**Figure 3:**
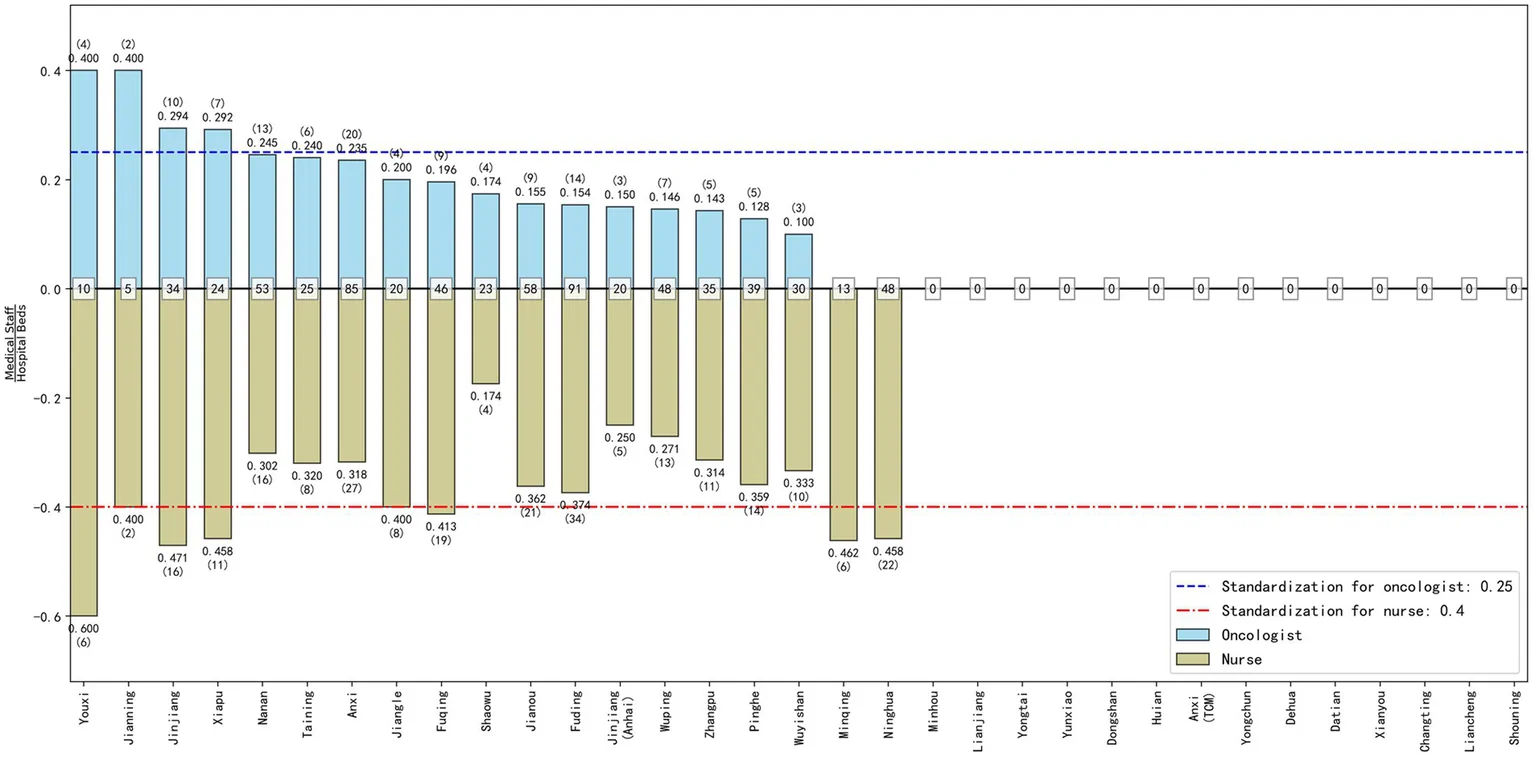
Oncologists and nurses versus beds in participating counties.

#### 3.1.3 Cancer-related diagnosis and treatment devices

Allocation of cancer-related medical devices in county-level hospitals was also investigated in our survey, and the results are illustrated in Table 2, where “1” and “0” denote the availability and nonavailability of corresponding medical devices, respectively. Table 2 shows that all 33 participating county-level hospitals have been equipped with X-ray machines, spiral CT scanners, MRI systems, gastrointestinal endoscopes, bronchoscopes, ECG machines, color Doppler ultrasound systems, and pathological microtomes. Moreover, 23 (69.7%), 7 (21.2%), and 8 (24.2%) county-level hospitals are equipped with immunohistochemistry equipment, molecular pathological detection equipment, and radiotherapy equipment, respectively. Only county-level hospitals in Fuqing, Minhou, Zhangpu, Huian, Anxi, Fuding, and Xiapu have equipped with molecular pathological detection equipment, while only county-level hospitals in Zhangpu, Jinjiang, Nanan, Anxi, Jiangle, Shaowu, Wuping, and Fuding have equipped radiotherapy equipment.

**Table 2:** Medical devices of participating county-level hospitals.

County-level hospital	X-ray	Spiral CT	MRI	Gastrointestinal Endoscope	Bronchoscope	ECG	Color Doppler Ultrasound	Pathological Microtome	Pathological Immunohistochemistry	Molecular Pathology Detection	Radiation Therapy
Fuqing	1	1	1	1	1	1	1	1	1	1	0
Minhou	1	1	1	1	1	1	1	1	1	1	0
Lianjiang	1	1	1	1	1	1	1	1	0	0	0
Minqing	1	1	1	1	1	1	1	1	0	0	0
Yongtai	1	1	1	1	1	1	1	1	0	0	0
Zhangpu	1	1	1	1	1	1	1	1	1	1	1
Yunxiao	1	1	1	1	1	1	1	1	1	0	0
Dongshan	1	1	1	1	1	1	1	1	1	0	0
Pinghe	1	1	1	1	1	1	1	1	0	0	0
Jinjiang	1	1	1	1	1	1	1	1	1	0	1
Jinjiang(Anhai)	1	1	1	1	1	1	1	1	1	0	0
Nanan	1	1	1	1	1	1	1	1	1	0	1
Huian	1	1	1	1	1	1	1	1	1	1	0
Anxi	1	1	1	1	1	1	1	1	1	0	1
Anxi(TCM)	1	1	1	1	1	1	1	1	1	1	0
Yongchun	1	1	1	1	1	1	1	1	0	0	0
Dehua	1	1	1	1	1	1	1	1	1	0	0
Ninghua	1	1	1	1	1	1	1	1	1	0	0
Jianing	1	1	1	1	1	1	1	1	1	0	0
Taining	1	1	1	1	1	1	1	1	0	0	0
Jiangle	1	1	1	1	1	1	1	1	1	0	1
Youxi	1	1	1	1	1	1	1	1	1	0	0
Datian	1	1	1	1	1	1	1	1	0	0	0
Xianyou	1	1	1	1	1	1	1	1	0	0	0
Shaowu	1	1	1	1	1	1	1	1	1	0	1
Wuyishan	1	1	1	1	1	1	1	1	1	0	0
Jianou	1	1	1	1	1	1	1	1	1	0	0
Wuping	1	1	1	1	1	1	1	1	1	0	1
Changting	1	1	1	1	1	1	1	1	1	0	0
Liancheng	1	1	1	1	1	1	1	1	0	0	0
Shouning	1	1	1	1	1	1	1	1	0	0	0
Fuding	1	1	1	1	1	1	1	1	1	1	1
Xiapu	1	1	1	1	1	1	1	1	1	1	0

### 3.2 Cancer-related comprehensive service capabilities in county-level hospitals

We investigated the comprehensive cancer-related service capabilities of all 33 participating county-level hospitals, including five categories: (1) prevention and screening, (2) diagnosis and treatment, (3) cancer nursing, (4) regional collaboration, and (5) cancer information platform. These five categories are further divided into more detailed subcategories, and the survey results are shown in the table heatmap of Figure 4, in which the dark-colored, light-colored, and blank grids denote full availability, partial availability, and nonavailability, respectively. Figure 4 is sorted by capability level from strong to weak. In the following sections, we illustrate cancer-related comprehensive service capabilities by category for all participating county-level hospitals.

**Figure 4:**
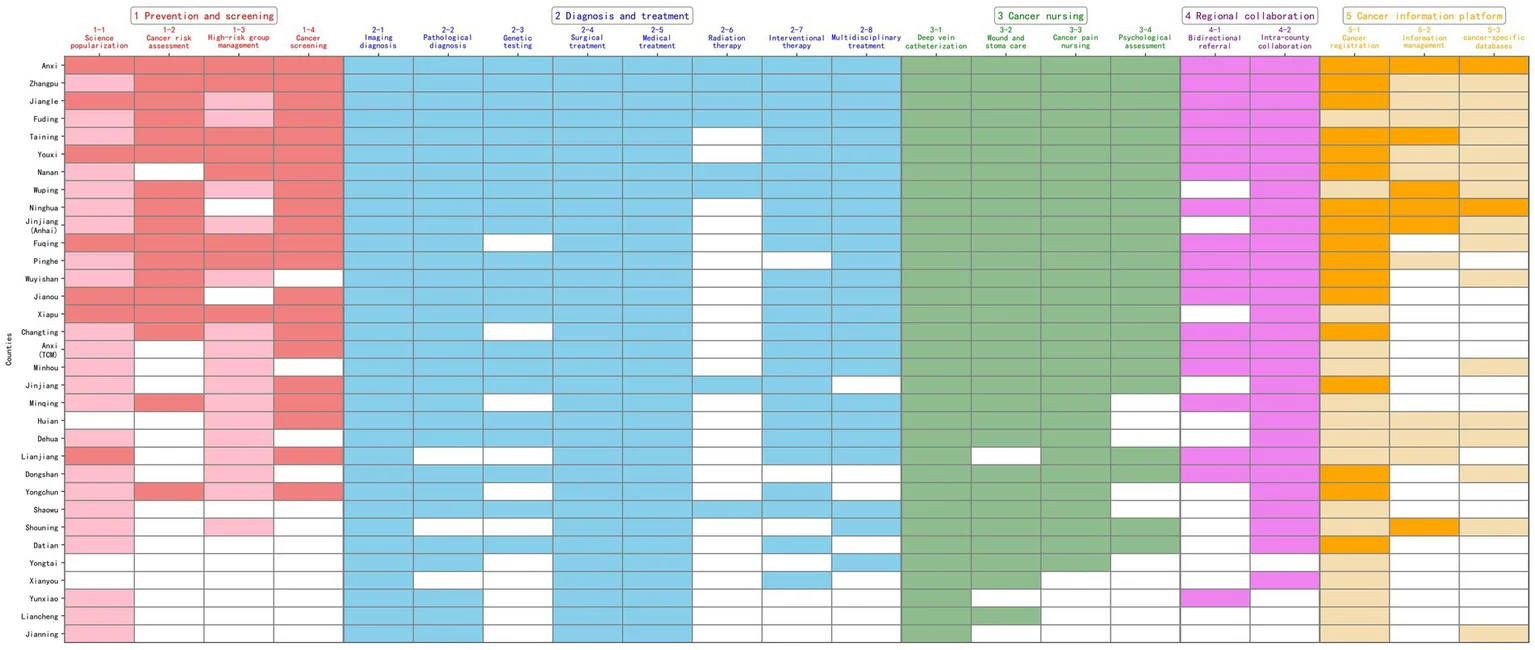
Cancer-related comprehensive service capabilities of all 33 participating county-level hospitals.

#### 3.2.1 Cancer prevention and screening ability

As shown in Figure 4, the capabilities of cancer prevention and screening include science popularization, cancer risk assessment, high-risk group management, and cancer screening. The statistical results are illustrated in Figure 5. Figure 5 reported that (a) 30 (91.9%) participating hospitals implement science popularization in cancer health care, but only 7 (21.2%) of them organize 20 popular science activities per year; (b) 17 (51.5%) participating hospitals carry out cancer risk assessment; (c) 24 (72.7%) participating hospitals have established mechanisms for high-risk group management, but 16 (48.5%) of them report inadequate execution, while 9 (27.3%) participating hospitals have no high-risk group management; and (d) 21 (63.6%) participating hospitals conduct cancer screening.

**Figure 5:**
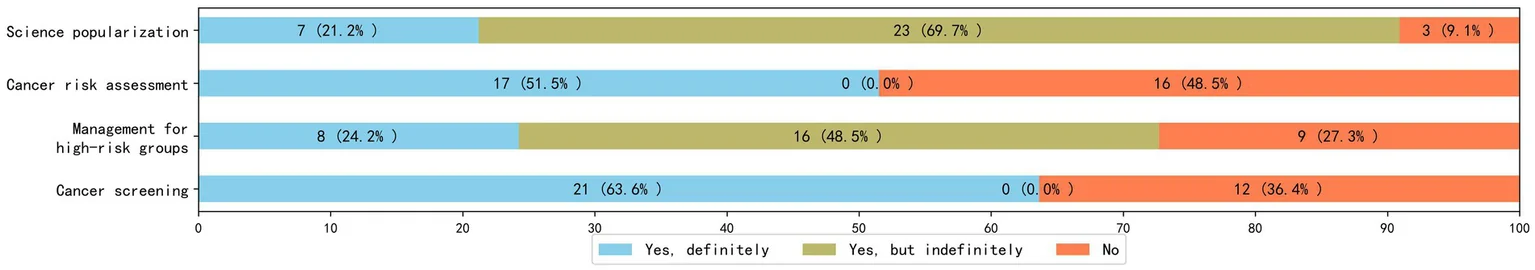
Cancer prevention and screening ability in county-level hospitals.

#### 3.2.2 Cancer diagnosis and treatment

As shown in Figure 4, the capabilities of cancer diagnosis and treatment in county-level hospitals include imaging diagnosis, pathological diagnosis, genetic testing, surgical treatment, medical treatment, radiation therapy, interventional therapy, and multidisciplinary treatment (MDT). The statistical results are illustrated in Figure 6. Figure 6 reported that (a) all 33 (100%) participating county-level hospitals are capable of performing regular diagnosis and treatment measures, such as imaging diagnosis, pathological diagnosis, medical treatment, and surgical treatment, owing to the necessary devices and personnel (see Table 2); and (b) 11 (33.3%), 25 (75.8%), and 7 (21.2%) participating hospitals are unable to conduct genetic testing, radiation therapy, and interventional treatment, respectively, owing to insufficient devices (also see Table 2). Besides, the detailed MDT cases for all participating county-level hospitals can be found in Table 3 in a descending order. Table 3 shows that 25 (75.8%) participating hospitals have established primary regulations and procedures of MDT with relatively stable medical teams to conduct MDT, whereas the remaining 8 (24.2%) still need progress.

**Figure 6:**
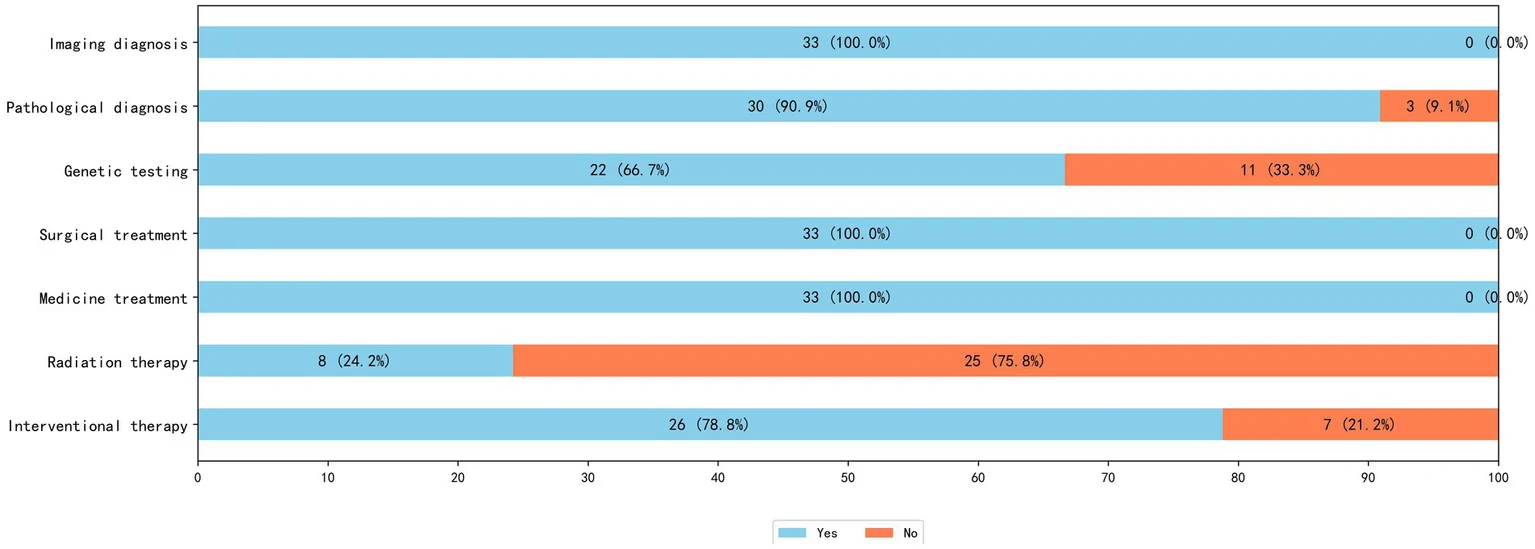
Cancers be diagnosed and treated in county-level hospitals.

**Table 3:** MDT cases of all participating county-level hospitals.

Hospital	Nanan	Fuqing	Anxi	Jianou	Wuyishan	Jiangle	Fuding	Taining	Dehua	Datian	Huian
MDT	974	841	395	261	236	207	135	40	37	36	35
Hospital	Ninghua	Pinghe	Changting	Yongtai	Wuping	Xianyou	Youxi	Zhangpu	Xiapu	Anxi(TCM)	Jinjiang(Anhai)
MDT	30	24	23	20	19	17	15	13	12	7	5
Hospital	Minhou	Minqing	Shouning	Yunxiao	Dongshan	Jinjiang	Yongchun	Jianning	Shaowu	Liancheng	Lianjiang
MDT	4	3	3	0	0	0	0	0	0	0	0

#### 3.2.3 Cancer nursing ability

Our survey focuses on several core competencies that are closely related to specialized cancer nursing and directly affect the treatment outcomes and quality of life of cancer patients, primarily to understand the extent to which county-level hospitals meet the specialized care needs of cancer patients. The capability of cancer nursing in county-level hospitals includes deep vein catheterization, wound and stoma nursing, cancer pain nursing, and psychological assessment, and the results are illustrated in Figure 7. Figure 7 shows that the first three capacities are basically well guaranteed in all participating county-level hospitals, with implementation rates of 100.0, 90.9, and 87.9%, respectively. However, the capacity for a psychological assessment, with a rate of 69.7%, remains insufficient to become a prominent shortcoming in specialized cancer nursing in county-level hospitals and is not conducive to realizing the “patient-centered” concept of whole-cycle cancer nursing.

**Figure 7:**
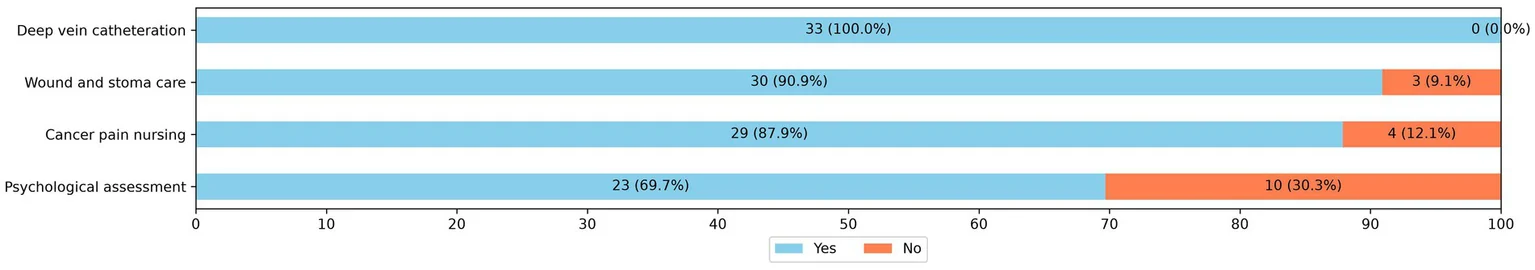
Capability of cancer nursing in county-level hospitals.

#### 3.2.4 Regional collaboration ability

Regional collaboration consists of bidirectional referrals and intracounty collaboration. Medical resources, especially for large-scale and high-end devices, are generally not equally available in all participating county-level hospitals (see Table 2). Thus, the bidirectional referral of cancer patients and hospital collaboration should be necessary for all county-level hospitals. Bidirectional referrals can be further divided into three subdimensions, namely, bidirectional referral regulations and workflows, regional collaborative management protocols, and at least one successful patient referral, whereas intracounty collaboration can be further divided into four subdimensions, namely, project guidance and quality control of cancer screening, technical training for routine diagnosis and treatment, online guidance and expert consultation, and joint management of high-risk groups and rehabilitation of cancer patients. The availability of the subdimensions for bidirectional referral and intracounty collaboration is listed in Table 4.

**Table 4:** Availability of regional collaboration in participating county-level hospitals.

Whether conducting following regional collaboration items?	Yes	No
1 Bidirectional referral	19 (57.6%)	14 (42.4%)
1–1 Bidirectional referral regulation and workflow	28 (84.8%)	5 (15.2%)
1–2 Regional collaborative management protocol	25 (75.8%)	8 (24.2%)
1–3 At least one patient referral	21 (63.6%)	12 (36.4%)
2 Intra-county collaboration	11 (33.3%)	22 (66.7%)
2–1 Project guidance and quality control of cancer screening	26 (78.8%)	7 (21.2%)
2–2 Technical training of routine diagnosis and treatment	24 (72.7%)	9 (27.3%)
2–3 Online guidance and expert consultation	20 (60.6%)	13 (39.4%)
2–4 Joint management of high-risk group and rehabilitation cancer patients	19 (57.6%)	14 (42.4%)

Table 4 reported that among the three subdimensions of bidirectional referral, 28 (84.8%) participating hospitals have set up bidirectional referral regulations and procedures, 25 (75.8%) participating hospitals have signed regional collaborative management protocols with at least one higher-level hospital, and 21 (63.6%) participating hospitals have successfully referred one cancer patient. In particular, 17 (51.5%) participating hospitals have successfully referred at least 10 cancer patients in recent years. Only 19 (57.6%) participating hospitals simultaneously fulfill all three subdimensions of bidirectional referral. Moreover, our survey revealed that 16 (48.5%) hospitals successfully referred fewer than 10 cancer patients or did not carry out bidirectional referrals in the past year, in other words, fewer than one case on average per month. This also indicates that the implementation of bidirectional referrals is far from ideal. The details of three subdimensions of bidirectional referral of participating county-level hospitals in Fujian province are listed in Table 5 in descending order of bidirectional cases. Table 5 suggests an obvious trend that county-level hospitals with a large amount of bidirectional referral cases always have established bidirectional referral regulation and procedures, as well as regional collaborative management protocols.

**Table 5:** Details of three subdimensions of bidirectional referral in county-level hospitals.

Hospital	Nanan	Anxi	Youxi	Fuding	Lianjiang	Changting	Fuqing	Wuyishan	Anxi(TMC)	Dongshan	Minqing
Bidirectional referral regulation and workflow	Yes	Yes	Yes	Yes	Yes	Yes	Yes	Yes	Yes	Yes	Yes
Regional collaborative management protocol	Yes	Yes	Yes	Yes	Yes	Yes	Yes	Yes	Yes	Yes	Yes
Bidirectional referral cases	721	669	443	434	288	186	155	117	78	60	40

Hospital	Jiangle	Taining	Ninghua	Zhangpu	Jianou	Minhou	Jianning	Liancheng	Pinghe	Yunxiao	Shaowu
Bidirectional referral regulation and workflow	Yes	Yes	Yes	Yes	Yes	Yes	No	No	Yes	Yes	Yes
Regional collaborative management protocol	Yes	Yes	Yes	Yes	Yes	Yes	Yes	Yes	Yes	Yes	Yes
Bidirectional referral cases	32	30	22	17	14	13	27	25	3	2	0

Hospital	Jinjiang(Anhai)	Yongchun	Xiapu	Jinjiang	Huian	Datian	Wuping	Shouning	Yongtai	Dehua	Xianyou
Bidirectional referral regulation and workflow	Yes	Yes	Yes	Yes	Yes	Yes	Yes	Yes	No	No	No
Regional collaborative management protocol	Yes	Yes	Yes	No	No	No	No	No	No	No	No
Bidirectional referral cases	0	0	0	0	0	0	0	0	0	0	0

Among the four subdimensions of intra-county collaboration, Table 4 shows that 26 (78.8%), 24 (72.7%), 20 (60.6%), and 19 (57.6%) participating hospitals have conducted project guidance and quality control for cancer screening, technical training for routine diagnosis and treatment, online guidance and expert consultation, and joint management of high-risk groups and rehabilitation of cancer patients, respectively. Only 11 (33.3%) participating hospitals simultaneously fulfill all four subdimensions of intracounty collaboration, and county-level collaboration is far from being achieved.

#### 3.2.5 Cancer information platform in county-level hospitals

Cancer information platform of county-level hospitals in our survey includes cancer registration, information management, and electronic health records and cancer-specific databases. Cancer information platform is important for the implementation of full-process management for cancer screening, diagnosis, treatment, recovery, and rehabilitation. An information platform would greatly increase the efficiency and ability of county-level hospitals to manage high-risk groups and cancer patients and serve as the fundamental infrastructure for establishing residents’ electronic health records and cancer-specific disease databases. In addition, an information platform would overcome geographic restrictions and optimize the collaborative management of intracounty cancer patients. The relevant statistical data from our survey are illustrated in Figure 8. Figure 8 reported that: (a) All participating hospitals have conducted cancer registration, but relayed reporting and underreporting are observed in 16 (48.5%) participating hospitals. (b) 6 (18.2%) participating hospitals have established a cancer information platform to share data with primary-level medical institutions within respective counties, but are unable to share data with hospitals in prefecture-level cities and province capital; 9 (27.3%) participating hospitals have established cancer information platform within respective hospitals, but are unable to share data with primary-level medical institutions within respective counties or hospitals in prefecture-level cities and provincial capital; while cancer information platform is still not available for 18 (54.5%) participating hospitals; all the figures above indicate a significant insufficiency of cancer information platform in county-level hospitals. (c) The nonavailability of 15 (45.5%) participating hospitals suggests that the electronic health records and cancer-specific databases are still in a preliminary stage.

**Figure 8:**
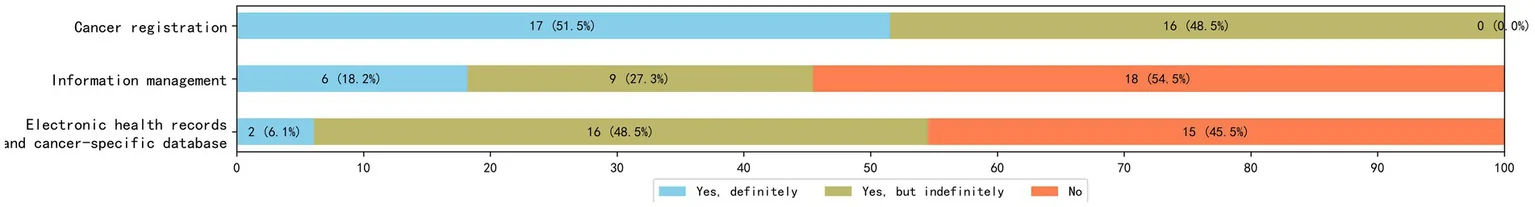
Cancer information platform level of county-level hospitals.

## 4 Discussion

The above survey results assess the current status of cancer-related medical resources and comprehensive service capabilities of county-level hospitals in Fujian Province and reveal certain insufficiencies in both aspects, which would predictably impact the standardization of cancer diagnosis and treatment. As the leading institution in county-level regions in the national hierarchical diagnosis and treatment system, the core orientation of county-level hospitals is to undertake primary consultation, diagnosis and treatment of regular diseases, and rehabilitation of stable patients and to serve as the linkage with higher-level hospitals via bidirectional referral. However, the dual weakness of medical resources and comprehensive service capacities of participating county-level hospitals would compromise the standardization of cancer diagnosis and treatment and suppress the realization of the four core purposes of the national hierarchical diagnosis and treatment system (first consultation at primary-level medical institutions, bidirectional medical referrals, separate treatment of acute and chronic diseases, and top-down and bottom-up linkages).

### 4.1 Insufficiency of medical resources reduces the quality of cancer diagnosis and treatment in county-level hospitals

The first resource insufficiency is cancer-specific in-patient beds. According to the China Health Statistics Yearbook 2023 (27), the quantity of cancer-specific in-patient beds in the whole nation is 279,644, and the number of cancer-specific beds per 1,000 people is 0.1981. Moreover, the number of cancer-specific beds in Fujian Province is 5,493, and the number of cancer-specific in-patient beds per 1,000 people is 0.1312 (5). Although the number of cancer-specific in-patient beds in Fujian Province gradually increased from 5,012 in 2020 to 5,493 in 2022, with an accelerating growth rate of 9.6% (17), which indeed exceeds the nationwide 8.07% growth, the total number of cancer-specific in-patient beds in Fujian Province is still significantly short for the 27th ranking in the whole nation. Figures 1, 2 together indicate that the participating county-level hospitals clearly experience a shortage of cancer-specific in-patient beds. All 33 participating hospitals involve cancer patients, but only 8 (24.2%) hospitals have set up independent oncology departments and wards and are thereby capable of centralized admission and treatment of cancer patients. The remaining 25 (75.8%) hospitals decentralize the admission and treatment of cancer patients in different departments, such as general surgery, respiratory medicine, and gastrointestinal medicine. Such decentralization inevitably results in low standardization for cancer diagnosis and treatment. Adding extra cancer-specific in-patient beds in a short time may be difficult due to constraints of limited funds, medical personnel, and wards. However, it is possible to modify some in-patient beds into cancer-specific ones for basic oncological inpatient care and palliative care, so that cancer-specific bed resources would be probably optimized with sufficient standardization for cancer diagnosis and treatment.

The second resource insufficiency is medical staff. The survey results in Figure 3 suggest a significant shortage of oncologists and oncology nurses in county-level hospitals in Fujian Province, even though oncologists and oncology nurses are unavailable for some county-level hospitals. Many medical staff in county-level hospitals have never received systematic and specific oncology training in higher-level hospitals. This shortage of cancer-specific diagnoses and treatments in county-level hospitals can lead to insufficient admission of cancer patients. In addition, provincial-level and top-tier municipal hospitals have strong siphoning effects on cancer patients (28). Both reasons lead to a corresponding low utilization of medical staff in nearby county-level hospitals (28). Thus, these nearby county-level hospitals would actively or passively reduce or even abandon the allocation of oncologists and oncology nurses, which would further intensify the cross-county tendency of patients and possibly jeopardize the standardization of cancer diagnosis and treatment. A similar medical staff insufficiency trend in rural area is also reported in America, while possible intervention strategies, such as fellowship training and hub-and-spoke mode, might be applied to slow down such a trend (29). In China, senior oncologists are regularly assigned to lower-level hospitals for guidance, which would also alleviate oncologist insufficiency in county-level hospitals.

The third resource insufficiency is cancer-specific devices. The device allocations in Table 2 suggest that the demand of primary diagnosis and treatment for local residents can be satisfied in the 33 participating county-level hospitals in 31 counties owing to the effect of the “Thousand County Project” (30), and the device allocation differences among counties are immunohistochemistry equipment, molecular pathological detection equipment, and radiotherapy equipment. However, the allocation of these cancer-specific devices generally requires a large amount of funding support; thus, it should be estimated according to the local population and health requirements. In other words, not every county-level hospital should be equipped with these devices. The corresponding insufficiency of precise, individualized, and comprehensive diagnosis and treatment to meet the higher demand for complex and complicated cancers may be compensated for by collaboration with higher-level hospitals and bidirectional referrals.

### 4.2 Insufficiency of comprehensive cancer-related service capabilities compromises survival prognosis for cancer patients

Currently, the comprehensive cancer-related service capabilities of hospitals tend toward a patient-centered mode, which requires effective interventions in the full-cycle stages of cancer patients (cancer prevention, screening, diagnosis, treatment, and rehabilitation) to increase the 5-year survival rate. The relatively lower survival rate in rural areas than in urban areas (6) suggests a severe reality of the defective cancer-related comprehensive service capacities of county-level hospitals. The survey data in Figures 4–8 of five subcategories of comprehensive service capacities of participating county-level hospitals are integrated into the radar map in Figure 9. Figure 9 reported that science popularization, high-risk group management, radiation therapy, cancer information platform, electronic health records and cancer-specific databases are particularly inadequate for cancer-related comprehensive service capacities of county-level hospitals.

**Figure 9:**
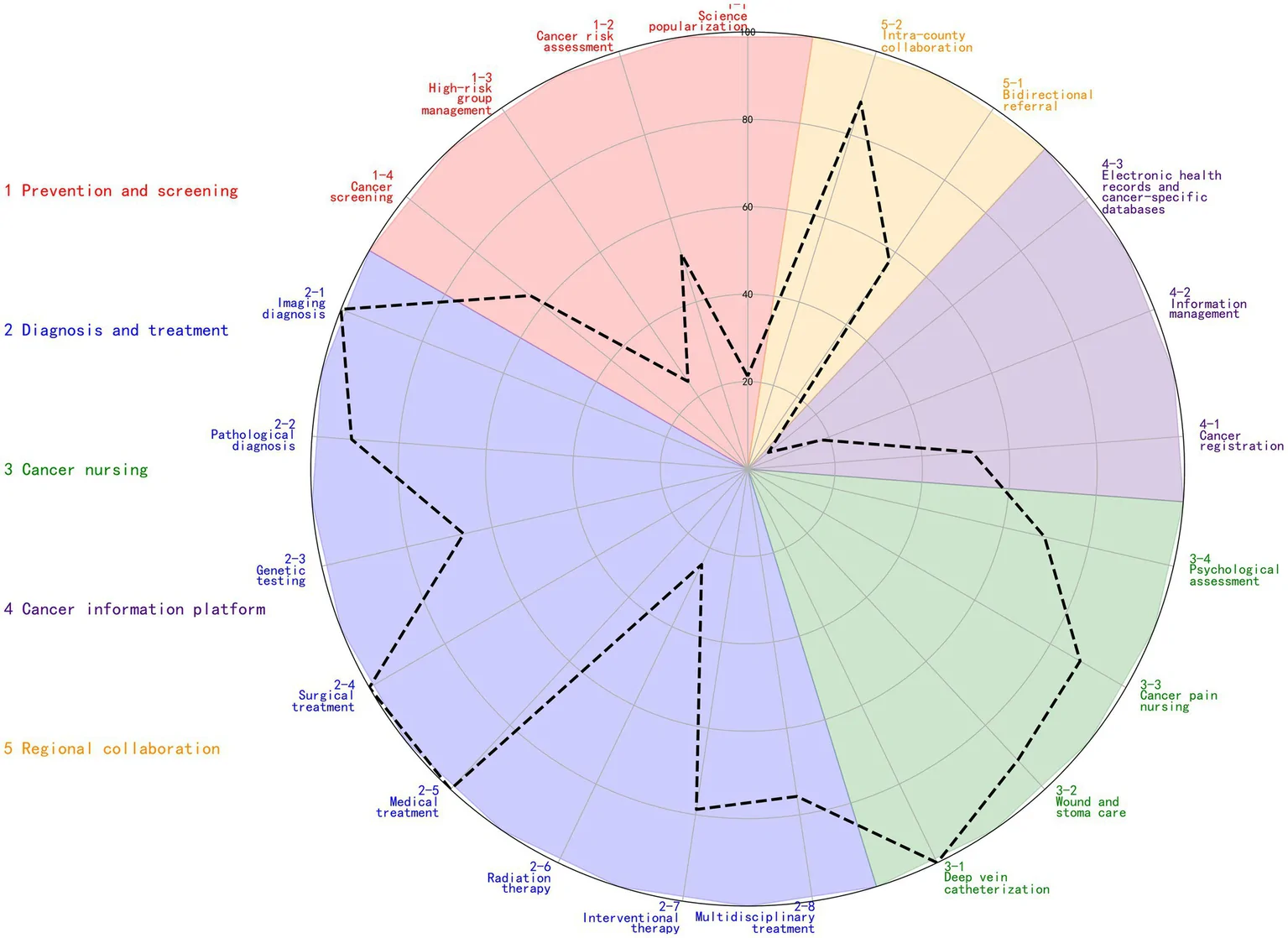
Radar map of cancer-related comprehensive service capabilities of participating county-level hospitals.

The first capacity insufficiency is cancer prevention and screening. In the category of cancer prevention and screening, sufficient science popularization would help rural residents establish a correct understanding of cancer-related diseases and corresponding effective diagnosis and treatment measures (31, 32). Our survey suggests that 23 of 33 (69.7%) participating county-level hospitals conduct fewer than 12 science popularization activities on cancer-related diseases per year, indicating less than 1 science popularization activity per month. Moreover, rural residents generally tend to avoid science popularization because of their fear of cancer. They believe that no discomfort is equal to good health and that a medical check-up is unnecessary. Several field studies suggest lower cancer literacy among rural residents in different sections of China due to a lack of cancer prevention popularization (33–35). A similar dilemma is also observed in America that the low literacy of high-incidence cancer, such as lung cancer, in rural areas is affected by low income, long distance to screening centers, lack of cancer prevention knowledge, insufficient insurance (36). Thus, influenced by the insufficient scientific population of medical institutions and their own misconceptions, knowledge of cancer prevention and treatment is not well understood or accepted by rural residents. In addition, 23 of 33 (69.7%) participating county-level hospitals provided feedback in our survey that the inadequate intensity of government policy and financial support prevented the initiatives of county-level hospitals from conducting sufficient and necessary cancer screening. Therefore, insufficient cancer screening may jeopardize the opportunity for early diagnosis and intervention in rural residents. Native dialect support and face-to-face would reduce reluctance of rural residents on science popularization to better evaluate cancer literacy (37). County-level hospitals and primary-level hospitals are encouraged to provide medical outreach services in rural areas and simultaneous guidance of screening appointment and registration.

The second capacity insufficiency is cancer diagnosis and treatment capacity. The insufficiency of cancer diagnosis and treatment capacity in county-level hospitals may cause patients to miss the golden window for effective cancer treatment. The insufficient diagnosis and treatment experience for medical staff of clinical, radiology, and pathology departments in county-level hospitals and the insufficient implementation of cancer staging strategies and lack of diagnostic devices, such as emission computed tomography and molecular pathology detection, lead to inaccurate imaging and pathological diagnosis and corresponding pretreatment TNM (Tumor Node Metastasis) cancer staging. In addition, our data in Figure 6 suggest that awareness of MDT is not sufficient among clinical oncologists in county-level hospitals and that no MDT mechanism is established in 3 of 33 (9.1%) participating hospitals, whereas no MDT team is established in 5 of 33 (15.2%) participating hospitals. Research in southwestern China also suggests a similar situation of insufficient radiotherapy equipment and MDT awareness (38). Cancer treatments in county-level hospitals still rely on a single clinical treatment method, and rash surgical regimens may exist without sufficient multidisciplinary discussions. For example, surgical resection plans were formulated in some county-level hospitals without any MDT discussion for cancer patients admitted to the general surgery department. For another example, in hospitals without radiation therapy equipment, comprehensive treatment formulation is compromised because of the absence of oncology radiation physicians. Regional MDT collaboration (39) is probably a feasible way to enhance diagnosis and treatment capabilities of county-level hospitals. A MDT in county-level hospital may consist of local oncologists and one or more senior oncologists of nearby higher-level hospitals to provide comprehensive assessment and standardized treatment plan for patients.

The third capacity insufficiency is inadequate implementation of bidirectional referral, which increases medical costs for cancer patients, reduces the efficiency of hospital resource utilization, and directly suppresses the construction of a hierarchical diagnosis and treatment system. To implement hierarchical diagnosis and treatment systems, standardized bidirectional referral regulations and procedures should be established along with a unified bidirectional referral information platform, such as an internet bidirectional referral system (40), and county-level hospitals should be able to directly apply and complete bidirectional referrals of cancer patients on such a platform. Besides, such a platform would also facilitate primary-level medical institutions to manage chronic cancer patients and patients with advanced cancers by regular follow-up and possible palliative care. However, the inter-regional referral regulations and procedures in Fujian Province are not uniform. Thus, bidirectional referral cannot be implemented with a unified standard, and corresponding surveillance cannot be effectively conducted. At present, bidirectional referral in Fujian Province remains a voluntary and dispersed practice. Moreover, an interest game to compete for patients and funds exists between higher-level hospitals and lower-level hospitals to weaken the initiative of bidirectional referral, and no stable cooperative relationships can be established. A primary provincial-level bidirectional referral platform has been started in Fujian Province; however, patient diagnosis and treatment information should be uploaded in document form on this platform causing heavy workloads. Such a complicated referral procedure would directly compromise referral efficiency. Hence, although this bidirectional referral platform seems to be accepted by most hospitals in Fujian Province, it is generally not used in practice. Moreover, the existing platform is unable to cover chronic cancer patients and patients with advanced cancers for home treatment and recovery. The impaired workflow of bidirectional referral would compromise the continuity of medical services and jeopardize the fulfillment of the functional roles of medical institutions at all levels to directly affect the quality of cancer diagnosis and treatment and increase medical costs. Patients with complex cancer cases cannot receive timely diagnosis and treatment from higher-level hospitals, nor can patients in stable stages return to nearby county-level hospitals or primary-level medical institutions. The regional hub-and-spoke mode in America (41) would possibly mitigate bidirectional referral dilemma in Fujian province. In such a mode, cancer patients may be estimated to be admitted in local county-level hospitals or to be transferred to higher−/lower-level medical institutions, so that the medical resources would be used in maximum efficiency, and cross-city healthcare seeking behavior may be greatly reduced.

The last capacity insufficiency is cancer information platform. Cancer information platform is the core process of full-cycle cancer management for cancer patients in county areas and involves the whole workflow of high-risk group management, cancer screening, diagnosis, treatment, rehabilitation, and follow-up. The insufficiency of cancer information platform of county-level hospitals in Fujian province would lead to the lack of electronic health record and cancer-specific database, which in turn causes that our survey could not convey accurate statistical data on cancer prevention, diagnosis, treatment, and nursing according to cancer types. Cancer information platform requires high-intensity continuous funding investment for software development, maintenance, and updates, as well as collaborative management of data and processes across multi-level institutions, such as county-level hospitals, town-level health centers, and public clinics. These primary hospitals are highly dependent on government funding, and most county-level hospitals are unable to bear the financial burden of the full-cycle cancer information platform. Our survey suggests that the insufficiency of the cancer information platform and feedback lag of data integration were reported in 25 of 33(75.7%) participating county-level hospitals. Insufficiency of cancer information platform measures would inevitably cause inadequate management of high-risk groups to miss opportunities for early diagnosis and treatment, and the corresponding medical information barricade would lead to repeated check-ups and unnecessary medical costs. Moreover, irregular follow-up caused by insufficient information jeopardizes the assessment of treatment effectiveness and the subsequent treatment schedule for cancer patients. The successful practice of Ohio Cancer Assessment and Surveillance Engine (42) with data sharing among Ohio Cancer Incidence Surveillance System, American Community Survey database, Health Resources and Services Administration, and Food and Drug Administration would greatly facilitate the full-life-cycle management, including cancer registry, screening, prevention, staging, diagnosis, treatment, research, and follow-up. Similar cancer information platform might be considered to be explored and built in Fujian province in a locally adapted approach.

### 4.3 Gradual development progress in cancer prevention and treatment centers to improve county-level cancer diagnosis and treatment capacities

As China is confronting the significant gap in medical service capacity between rural and urban regions, the Chinese government endeavors to promote comprehensive service capacities of county-level hospitals, system development of hierarchical diagnosis and treatment, and the inclination of high-quality medical resources at the provincial and municipal levels to county-level regions so that county-level hospitals are fully able to undertake the diagnosis and treatment of common and frequently occurring diseases for county-level residents. Nevertheless, more efforts should be necessary on the basis of an optimized referral rate design and investigation (43). Under such circumstances, the “Thousand County Project” was launched in 2021 (30), and 1,233 county-level hospitals were selected throughout the nation as construction units after a certain capacity assessment (24). Thirty-one out of 33 participating county-level hospitals are on the list, while the remaining two volunteered to participate in our survey. From 2021 to 2024, the medical service capabilities of these 31 participating county-level hospitals in our survey were effectively enhanced, their emergency care and first aid capabilities further improved, and the sharing of county-level medical resources and the level of high-quality management continuously increased.

However, the cancer diagnosis and treatment resources and capabilities of these 33 participating county-level hospitals still have insufficiencies in different aspects. Therefore, Fujian Province in 2024 launched a development project of county-level cancer prevention and treatment centers based on the “Thousand County Project” (30), aiming at consolidating the regional cancer prevention and treatment network and promoting cancer treatment standardization. A county-level cancer prevention and treatment center takes county-level hospitals as the core and integrates primary-level medical institutions such as township health centers and village clinics within the county to realize hierarchical medical care by means of resource sharing and division of labor and collaboration and to provide continuous and efficient cancer diagnosis and treatment services for residents in the county. Combined with the survey data on the cancer diagnosis and treatment capabilities of the 33 participating county-level hospitals, we found that some hospitals are relatively mature in terms of cancer prevention and screening, diagnosis and treatment, cancer-specific nursing, regional collaboration, and cancer information platform. To rationally plan the input scale of medical resources, priority should be given to addressing the shortcomings and weaknesses of these hospitals to further enhance their cancer diagnosis and treatment capacities, especially in primary-level medical institutions (2), so that cancer patients are able to have access to first consultation at primary-level medical institutions and rehabilitation at such institutions. With inclined medical resource input, these hospitals will likely become the benchmarks of the remaining county-level hospitals to ultimately promote the genuine implementation of the hierarchical medical system in the oncology field and to improve the early diagnosis rate of major cancer types as well as the 5-year survival rate.

### 4.4 Limitation

Although our survey provides concrete data on cancer-related medical resources and comprehensive service capabilities among county-level hospitals, limitations should be disclosed. The first limitation is that the hospitals included in our survey were not randomly selected but rather were selected on the basis of the “Thousand County Project” menu (30). Thus, the samples in our survey are limited in that they cover only approximately 60% of the counties in Fujian Province. The second limitation is that only medical personnel and devices are summarized, while no further specific data on different cancer types are provided, including cancer prevention, screening, diagnosis, and treatment. The third limitation is that our study is not related to the possible influence of medical costs and insurance policies on the cancer diagnosis and treatment orientation of county-level hospitals in Fujian Province. In other words, our study may be limited in its ability to analyze the fundamental impact of medical pricing policies, medical insurance policies, and pharmaceutical policies on the imbalanced allocation and insufficient accessibility of cancer-specific medical resources in county-level hospitals in Fujian Province precisely. These limitations should be explored in future studies.

## 5 Conclusion

This paper estimates the current status of cancer-specific medical resources and comprehensive service capabilities at selected county-level hospitals in Fujian Province, southeastern China. The results reveal an uneven distribution of medical resources and insufficient service capabilities among these county-level hospitals. The fndings provide baseline data for further county-oriented allocation of high-quality medical resources and targeted service capacity improvement aiming at gradual pooling and integration of medical resources. More importantly, these baseline data serve as reference benchmarks for the subsequent assessment of further medical development and reformation in county-level hospitals. On the basis of these data, a dynamic tracing mechanism for variations in cancer-specific medical resources and medical service quality can be correspondingly established through data surveillance, effectiveness estimation, and iterative optimization to achieve closed-loop management. The ultimate goal is to guarantee assessable and convenient cancer diagnosis and treatment services for county residents and to provide solid decision support for alleviating the burden of cross-regional medical care.

## Data Availability

The data that support the findings of this study are available from the first author upon reasonable request.
